# Rickettsial Disease Outbreak, Mexico, 2022

**DOI:** 10.3201/eid2909.230344

**Published:** 2023-09

**Authors:** Ricardo J. Estrada-Mendizabal, Oscar Tamez-Rivera, Emelina Hinojosa Vela, Paulina Blanco-Murillo, Cordelia Alanis-Garza, Jaime Flores-Gouyonnet, Jessica Suhail Sauceda Garza, Gloria Yolanda Carranza Medina, Lilia Elida García Rodriguez, Alma Rosa Marroquin Escamilla

**Affiliations:** Tecnologico de Monterrey, Escuela de Medicina y Ciencias de la Salud, Monterrey, Mexico (R.J. Estrada-Mendizabal, O. Tamez-Rivera, P. Blanco-Murillo, C. Alanis-Garza);; Secretary of Health of Nuevo Leon, Monterrey (E. Hinojosa Vela, J.S. Sauceda Garza, G.Y. Carranza Medina, L.E. Garcia Rodriguez, A.R. Marroquin Escamilla);; Autonomous University of San Luis Potosi, San Luis Potosi, Mexico (J. Flores-Gouyonnet)

**Keywords:** rickettsial disease, outbreak, Mexico, 2022, rickettsia, Nuevo Leon, ticks, vector-borne infections, surveillance, bacteria

## Abstract

Beginning in 2022, Nuevo Leon, Mexico, experienced an outbreak of rickettsioses that is still ongoing despite multidisciplinary control efforts. A total of 57 cases have been confirmed, particularly affecting children. We report a high mortality rate among hospitalized persons in Nuevo Leon. Continuing efforts are required to control the outbreak.

Rickettsioses are life-threatening vectorborne infections transmitted by several arthropods, such as ticks, lice, fleas, and mites (*1*,*2*). Rickettsial diseases are an emerging threat in Mexico, particularly in the northern regions, where previous outbreaks have been reported (*3*). In 2022, the local epidemiologic surveillance department reported 57 confirmed and >500 probable rickettsial disease cases in Nuevo Leon, a semiarid state in northeast Mexico. This unprecedented and alarming increase represents the highest number of rickettsial disease cases in a single year in this region, showing significant contrast with 2021, when only 13 confirmed cases were reported. Although surveillance and preventive measures are continuously in place, additional multidisciplinary strategies were established after the outbreak was declared in May 2022.

The Mexican Institute of Epidemiology defines a probable case of rickettsiosis as a patient with fever and ≥2 compatible clinical and laboratory signs. Technicians at the State Laboratory of Public Health of Nuevo Leon perform real-time PCR targeting the *gltA* gene on all probable cases identified <7 days after symptom onset. A positive PCR result requires the presence of a well-defined sigmoid curve, where the 3 PCR-reaction phases are distinguished, plus a quantification cycle value ≤38. State laboratory staff use an indirect immunofluorescence antibody assay to analyze all samples collected 7–14 days after symptom onset and confirm cases through real-time PCR or, retrospectively, with seroconversion by immunofluorescence antibody analysis (*4*). The data discussed in this report comprise all 57 confirmed cases in 2022.

Compared with results from 2021, the incidence rate of rickettsioses in Nuevo Leon in 2022 rose from 0.2 to 0.9 cases/100,000 inhabitants. Most cases occurred in October (n = 14) and December (n = 9). The median patient age was 10 years (range 1–61 years); 59.6% of case-patients were female and 40.4% male. The pediatric population (≤18 years of age) represented 77% of all cases (Appendix Table). Most patients required hospitalization (n = 50), and all had a positive history of tick exposure within 2 weeks before symptom onset. More than half of cases (54%) originated in 2 remote municipalities of Nuevo Leon, where most patients had a positive contact history with stray dogs or cats. The most frequent clinical signs were fever (100%), petechial rash (56%), and tachycardia (40%) (Table). Predominant symptoms were headache (75%), abdominal pain (75%), myalgia (74%), and arthralgia (58%). Laboratory findings at hospital admission included anemia in 66% of case-patients, thrombocytopenia in 98% (median platelet count 25 × 10^3^*/*μL), leukocytosis in 35%, and leukopenia in 8%. 

**Table:** Clinical and paraclinical characteristics of patients with rickettsioses during outbreak in Neuvo Leon, Mexico, 2022

Characteristic	Total, n = 57	No. (%) patients		p value†
		Cured, n = 21	Died, n = 36	
Patient age, y				0.106
<4	8	1 (1.7)	7 (12.2)	
4–12	28	12 (21)	16 (28)	
13–18	8	1 (1.7)	7 (12.2)	
>18	13	7 (12.2)	6 (10.5)	
Patient sex				0.768
F	34	12 (21)	22 (38.5)	
M	23	9 (15.7)	14 (24.5)	
Clinical signs‡	n = 48	n = 14	n = 34	
Anemia				**0.012**
Yes	32	11 (22.9)	21 (43.7)	
No	16	3 (6.2)	13 (27)	
Thrombocytopenia				**0.007**
Yes	47	13 (27)	34 (70.8)	
No	1	1 (2)	0	
Leukocytosis				**0.01**
Yes	17	3 (6.2)	14 (29.1)	
No	31	11 (22.9)	20 (41.6)	
Leukopenia				**0.014**
Yes	4	2 (4.1)	2 (4.1)	
No	44	12 (25)	32 (66.6)	
Treatment with doxycycline				0.074
Yes	52	21 (36.8)	31 (54.3)	
No§	5	0	5 (8.7)	
Time to treatment initiation, h	n = 52	n = 21	n = 31	**0.007**
≤24	4	4 (7.6)	0	
>24	48	17 (32.6)	31 (59.6)	

*n values within columns indicate number of patients in category. †By χ^2^ test. Bold indicates statistical significance. ‡Anemia: female, hemoglobin <11.6 g/dL; male, hemoglobin <13.2 g/dL). Thrombocytopenia: female, platelets <157 × 10^9^/L; male, platelets <135 × 10^9^/L. Leukocytosis: leukocytes >9.6 × 10^9^ cells/L. Leukopenia: leukocytes <3.4 × 10^9^ cells/L. §Five patients died before treatment and had their diagnosis confirmed by autopsy.

Of the 57 case-patients, 52 were treated with doxycycline; the remaining 5 died before treatment and had their infections diagnosed through autopsy. The median time-to-treatment initiation from symptom onset was 4 days, and only 8% of the patients received prompt antibiotic therapy within the first 24 hours of symptom onset. More than half (63%) of the total case-patient population died, and median time from symptom onset to death was 5 days (range 2–17); median length of hospital stay was 1 day (range 0–41). The annual rickettsiosis mortality rate for the region was 0.6 deaths/100,000 inhabitants. 

To determine clinical, laboratory, and demographic associations with mortality, we performed χ^2^ testing by using SPSS Statistics software (IBM, https://www.ibm.com). We found statistically significant associations with mortality in patients with anemia (p = 0.012), thrombocytopenia (p = 0.007), leukocytosis (p = 0.01), and leukopenia (p = 0.014) at hospital admission. Likewise, a time-to-treatment initiation of 24 hours was associated with survival (p = 0.007). Among the 57 cases, 4 were confirmed as spotted fever group rickettsiosis because of seroconversion to *Rickettsia rickettsii* antigens, 5 seroconverted to *R. typhi* and were confirmed as typhus group rickettsiosis, and the remaining 48 cases were tested by molecular analysis and were confirmed as simply rickettsiosis (*Rickettsia* sp.)—that is, PCR did not discriminate between spotted fever group and typhus group rickettsiae.

An alarming feature of this ongoing outbreak is its high fatality rate (63%). The most recent outbreaks of rickettsiosis in Mexico reported fatality rates of 40% in Sonora and 29% Baja California (*5*,*6*). In northeastern Mexico, the brown dog tick (*Rhipicephalus sanguineus*) is highly prevalent (Figure), posing a high risk for rickettsioses (*7*). Social determinants of health in hard-to-reach municipalities are thought to contribute to the rise in rickettsial disease cases. The abundance of stray animals, lack of healthcare accessibility, and poor disease knowledge may play a significant role in this outbreak. 

**Figure:**
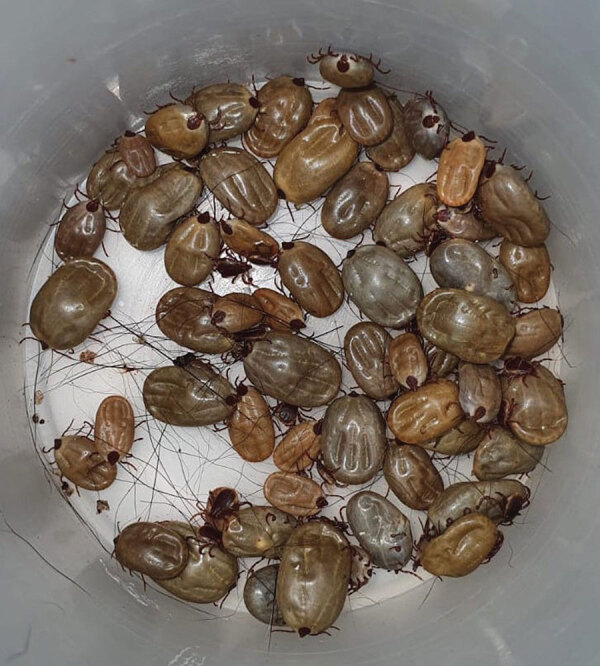
Brown dog ticks collected by the vector control department of Nuevo Leon, Mexico, in a hard-to-reach municipality.

To date, the local epidemiologic surveillance department has led various interventions in an attempt to control the outbreak by implementing vector control strategies, educating healthcare personnel of high-risk municipalities, designating community champions against rickettsioses, and raising public awareness through media. Clinicians on the Mexico–United States border should have a high index of suspicion of rickettsiosis among febrile patients and consider early empiric antibiotic treatment to reduce mortality risk.

Appendix More information about rickettsial disease outbreak, Mexico, 2022.
