# Supplementary material for: Rickettsial Disease Outbreak, Mexico, 2022
Source: Emerg Infect Dis. 2023 Sep;29(9):1944–7. doi: 10.3201/eid2909.230344 (PMC10461685; doi:10.3201/eid2909.230344)
Supplement: Appendix — More information about rickettsial disease outbreak, Mexico, 2022. [file 23-0344-Techapp-s1.pdf]

Article DOI: http://doi.org/10.3201/eid2909.230344

EID cannot ensure accessibility for supplementary materials supplied by authors. Readers who have difficulty accessing supplementary content should contact the authors for assistance.

Rickettsial Disease Outbreak, Mexico, 2022

Appendix

Appendix Table. Demographic, clinical, and paraclinical characteristics of confirmed cases of rickettsiosis in Nuevo Leon, 2022.

Case	Age (y)	Sex	Main clinical signs									Sample	Test (PCR† or IFA)	IFA titers (IgG)		Bacteria	Final Outcome
			Fever	Headache	Myalgia	Arthralgia	Rash	Nausea	Vomiting	Abdominal pain	Tachycardia			Acute	Convalescence		
1	61	F	*	*	*	*	Pet	*	*	*		Serum	IFA	1:64	1:32,768	*R. rickettsii*	Cured
2	15	F	*	*	*	*	Pet/Pu	*	*	*	*	Whole blood	PCR			*R. species*	Death
3	37	F	*	*	*	*	Pet/Pu				*	Whole blood	PCR			*R. species*	Death
4	25	M	*	*	*				*	*		Spleen biopsy	PCR			*R. species*	Death
5	10	F	*	*	*	*				*		Whole blood	PCR			*R. species*	Death
6	8	M	*	*	*	*	Pet	*	*	*	*	Whole blood	PCR			*R. species*	Death
7	9	F	*	*	*	*			*	*		Spleen biopsy	PCR			*R. species*	Death
8	40	M	*		*	*	Mac/Pet	*	*			Serum	IFA	1:2,048	1:8,192	*R. typhi*	Cured
9	11	F	*	*	*	*						Serum	IFA	<1:64	1:2,048	*R. typhi*	Cured
10	6	F	*	*	*	*	Mac					Serum	IFA	1:512	1:8,192	*R. typhi*	Cured
11	56	F	*	*	*	*		*	*	*		Serum	IFA	1:4,096	1:32,768	*R. typhi*	Cured
12	13	M	*	*	*			*	*	*		Serum	IFA	1:64	1:16,384	*R. typhi*	Cured
13	9	M	*		*	*	Mac	*	*			Whole blood	PCR			*R. species*	Death
14	10	F	*				Pet	*	*	*	*	Whole blood	PCR			*R. species*	Death
15	5	M	*	*			Pet			*		Whole blood	PCR			*R. species*	Death
16	7	F	*	*			Mac/Pet			*		Whole blood	PCR			*R. species*	Cured
17	10	M	*	*	*			*		*		Whole blood	PCR			*R. species*	Cured
18	46	M	*	*						*		Whole blood	PCR			*R. species*	Cured
19	37	M	*		*	*	Mac/Pet				*	Whole blood	PCR			*R. species*	Death
20	3	M	*				Mac/Pet			*	*	Whole blood	PCR			*R. species*	Death
21	13	F	*	*	*	*	Pu	*	*	*	*	Whole blood	PCR			*R. species*	Death
22	17	F	*	*				*	*	*		Whole blood	PCR			*R. species*	Death
23	6	M	*	*						*		Whole blood	PCR			*R. species*	Cured
24	7	F	*				Mac					Whole blood	PCR			*R. species*	Cured
25	23	F	*	*	*	*	Mac/Pet					Whole blood	PCR			*R. species*	Cured
26	2	F	*				Mac/Pu/Pet		*	*	*	Whole blood	PCR			*R. species*	Death
27	6	M	*		*	*	Mac/Pet	*		*	*	Whole blood	PCR			*R. species*	Death
28	12	F	*	*	*	*			*	*	*	Whole blood	PCR			*R. species*	Death

Case	Age (y)	Sex	Main clinical signs									Sample	Test (PCR† or IFA)	IFA titers (IgG)		Bacteria	Final Outcome
			Fever	Headache	Myalgia	Arthralgia	Rash	Nausea	Vomiting	Abdominal pain	Tachycardia			Acute	Convalescence		
29	14	M	*	*	*	*	Mac/Pu/Pet	*	*	*		Whole blood	PCR			*R. species*	Death
30	3	F	*	*	*	*		*		*	*	Whole blood	PCR			*R. species*	Death
31	12	F	*	*	*		Mac/Pet					Whole blood	PCR			*R. species*	Death
32	4	F	*	*	*	*	Pet			*		Whole blood	PCR			*R. species*	Cured
33	3	F	*	*	*	*	Mac/Pet	*	*	*		Whole blood	PCR			*R. species*	Cured
34	5	M	*	*			Mac/Pu/Pet				*	Whole blood	PCR			*R. species*	Cured
35	61	F	*	*	*	*	Mac	*	*	*		Whole blood	PCR			*R. species*	Death
36	1	M	*				Pet	*	*	*	*	Whole blood	PCR			*R. species*	Death
37	16	M	*	*	*	*	Mac/Pet/Pu	*	*	*	*	Whole blood	PCR			*R. species*	Death
38	8	F	*		*	*	Mac/Pet/Pu			*	*	Whole blood	PCR			*R. species*	Cured
39	3	M	*		*		Pet	*	*	*		Whole blood	PCR			*R. species*	Death
40	18	F	*	*	*	*					*	Whole blood	PCR			*R. species*	Death
41	7	F	*	*	*		Mac/Pet		*	*		Whole blood	PCR			*R. species*	Death
42	5	F	*				Mac/Pet			*		Serum	IFA	<1:64	1:32,768	*R. rickettsii*	Cured
43	10	F	*	*			Mac/Pu	*	*	*	*	Whole blood	PCR			*R. species*	Death
44	31	F	*	*					*	*	*	Whole blood	PCR			*R. species*	Death
45	6	M	*	*	*	*	Mac/Pet	*	*	*	*	Whole blood	PCR			*R. species*	Death
46	3	F	*		*	*	Mac/Pet			*		Whole blood	PCR			*R. species*	Death
47	50	F	*	*	*	*				*		Whole blood	IFA	1:64	1:256	*R. rickettsii*	Cured
48	37	M	*	*	*	*			*	*		Serum	IFA	1:64	1:256	*R. rickettsii*	Cured
49	12	M	*	*	*		Pet	*	*			Whole blood	PCR			*R. species*	Death
50	8	M	*	*	*		Pet	*	*	*		Whole blood	PCR			*R. species*	Death
51	1	F	*				Pet	*	*	*		Whole blood	PCR			*R. species*	Death
52	7	M	*	*	*	*	Mac	*	*		*	Whole blood	PCR			*R. species*	Cured
53	41	F	*	*	*	*	Mac/Pet		*	*	*	Whole blood	PCR			*R. species*	Death
54	13	F	*	*	*		Mac/Pet/Pu				*	Whole blood	PCR			*R. species*	Death
55	10	M	*	*	*	*		*		*		Whole blood	PCR			*R. species*	Cured
56	8	F	*	*	*	*	Mac		*	*		Whole blood	PCR			*R. species*	Death
57	7	F	*	*	*	*	Mac/Pet	*	*	*	*	Whole blood	PCR			*R. species*	Death

†Whole genome sequence (WGS) was performed on all patients with a positive RT-PCR.
* = present; blank = absent; Mac = macular; Pu = purpuric; Pet = petechial; PCR = polymerase chain reaction; IFA = immunofluorescence assay; IgG = immunoglobulin G
